# Case Report: Probable paroxysmal sympathetic hyperactivity during vascular and postoperative critical illness with bilateral white matter abnormalities

**DOI:** 10.3389/fnins.2026.1935805

**Published:** 2026-10-29

**Authors:** Mozhen Li, Yujie Wei, Zhenhua Cai, Lusheng Wang, Shengwen Guo, Jifei Cao, Heng Tang, Lei Wang

**Affiliations:** 1 Intensive Care Unit, Wanbei Coal Electric Group General Hospital, Suzhou, Anhui, China; 2 Central Laboratory, Wanbei Coal Electric Group General Hospital, Suzhou, Anhui, China

**Keywords:** case report, critical illness, encephalopathy, paroxysmal sympathetic hyperactivity, white matter abnormalities

## Abstract

**Background:**

Recognition of paroxysmal sympathetic hyperactivity (PSH) during vascular and postoperative critical illness is complicated by overlapping infection and seizure features.

**Case presentation:**

A 37-year-old man developed encephalopathy and rigidity after extensive arterial thrombosis, thrombectomy, and suspected postoperative sepsis. January 2025 observations documented recurrent, transient, simultaneous tachycardia, increased blood pressure, tachypnea, fever, sweating, and abnormal posturing. The treating team diagnosed PSH, which remained among the February 12 transfer diagnoses. Brain magnetic resonance images dated February 20, 2025, showed symmetric bilateral periventricular and deep white matter abnormalities without definite diffusion restriction. Targeted serum and cerebrospinal fluid antibody testing was negative. A clinician-completed PSH Assessment Measure (PSH-AM) recorded 24 points; conservative reassessment retained recorded severity grades and yielded 20, still classified as probable PSH. Paroxysms persisted as documented infection improved, decreased during multimodal treatment, and recurred during combined medication reduction. At the February 12 intrahospital rehabilitation transfer, communication and tone had improved, but contractures and severe limb motor impairment persisted. Cerebral etiology remained uncertain, and seizures could not be excluded because electroencephalographic event capture was unavailable.

**Conclusion:**

The case highlights serial clinical recognition of probable PSH during vascular and postoperative critical illness, alongside infection assessment and attention to persistent rehabilitation needs.

## 1 Introduction

Paroxysmal sympathetic hyperactivity (PSH), also known as sympathetic storming, is a severe syndrome of episodic autonomic and motor dysregulation characterized by recurrent tachycardia, hypertension, tachypnea, hyperthermia, diaphoresis, and abnormal motor posturing following acquired brain injury (Jafari et al., 2022; Scott and Rabinstein, 2020). PSH is associated with acquired brain injury of both traumatic and non-traumatic etiologies, including traumatic brain injury, hypoxic–ischemic encephalopathy, intracranial hemorrhage, ischemic stroke, central nervous system infection, and other structural or functional cerebral insults (Louraoui et al., 2022; Moffet et al., 2026). Current pathophysiological models propose that disruption of descending inhibitory pathways and subsequent disinhibition of sympathetic and motor excitatory circuits contribute to recurrent episodes of excessive sympathetic activation.

Despite increasing clinical recognition, PSH remains underdiagnosed and may be confused with sepsis, epileptic seizures, neuroleptic malignant syndrome, malignant hyperthermia, tetanus, withdrawal syndromes, or severe spasticity because of substantial overlap in their clinical manifestations. Delayed recognition may contribute to prolonged intensive care unit (ICU) stay, increased metabolic demand, secondary complications, greater morbidity, and poor functional outcomes. Although the PSH Assessment Measure (PSH-AM) has improved diagnostic standardization, the diagnosis remains challenging in critically ill patients with complex systemic disease, fluctuating neurological manifestations, or nonspecific neuroimaging abnormalities (Chahar et al., 2026; Datta et al., 2025).

Systemic inflammation and infection can complicate interpretation of autonomic abnormalities in critically ill patients (Salasky et al., 2024). White matter signal abnormalities may further broaden the differential diagnosis without defining the cause of the paroxysmal syndrome. Clinical observations, episode-specific assessment, and investigation of competing conditions therefore need to be reported separately from mechanistic interpretation.

We report probable PSH recognized by the treating team during vascular and postoperative critical illness, with encephalopathy and bilateral white matter abnormalities. Repeated autonomic–motor episodes, multimodal management, and persistent motor disability illustrate diagnostic and rehabilitation challenges in this setting. Cerebral etiology and possible coexisting seizures remained unresolved.

## 2 Case presentation

A 37-year-old man was admitted on January 2, 2025, with approximately 1.5 months of limb rigidity after prolonged critical illness. No previous chronic neurological disease, epilepsy, or head trauma was reported. The admission history also recorded an unspecified cardiac condition since childhood without regular treatment and longstanding alcohol consumption, described as approximately 200 g of beverage daily. Beverage strength and last intake were not recorded; the history does not establish an ethanol dose or withdrawal diagnosis.

According to the retrospective January admission history, left leg pain and claudication began on September 20, 2024, and a local hospital diagnosed arterial embolism and prescribed unspecified oral medication. On November 4, investigations at our hospital identified abdominal aortic thrombosis. He was subsequently referred to another hospital and underwent lower-extremity thrombectomy on November 12. Primary vascular, procedural, and acute critical illness records from that earlier admission were unavailable for this review.

The admission history dates fever, altered consciousness, and generalized rigidity to November 14, 2 days after thrombectomy. The thrombolytic catheter and sheath were removed and ICU care, anti-infective treatment, and anticoagulation were provided for suspected postoperative sepsis. The infection source, cultures, antimicrobial regimen, and contemporaneous organ dysfunction and Sequential Organ Failure Assessment (SOFA) components could not be reconstructed. November 14 is therefore a retrospectively reported onset of neurological dysfunction, not a confirmed brain-injury date or the onset of a fully characterized PSH syndrome.

During the outside ICU admission, coma, upward eye deviation, and seizure-like motor events were reported; clinicians considered central nervous system (CNS) demyelination and symptomatic epilepsy. Original neurological assessment and electroencephalography (EEG) reports were unavailable. Continuous EEG during the paroxysmal events was unavailable, and no routine/intermittent EEG report or electrographic capture of a typical event could be recovered. On November 26, abdominal CT reportedly demonstrated bowel perforation, followed by exploratory laparotomy and ileostomy.

Consciousness gradually improved before the January 2 admission, but severe rigidity persisted. Table 1 summarizes the retrospectively reported outside-hospital course and dated inpatient observations.

**TABLE 1:** Clinical timeline and sources of evidence.

Date	Clinical events	Assessment and management	Source and limitations
20 Sep / 4 Nov 2024	Left leg pain and claudication with reported arterial embolism; abdominal aortic thrombosis identified on 4 Nov.	Unspecified oral treatment, then referral for vascular intervention.	January admission history; primary vascular records unavailable.
12 Nov 2024	Lower-limb arterial thrombectomy reported.	Vascular intervention at another hospital.	Retrospective admission history; procedural report unavailable.
14 Nov 2024	Fever, altered consciousness and generalized rigidity after thrombectomy.	Catheter/sheath removal, ICU care, anti-infective treatment and anticoagulation for suspected postoperative sepsis.	Retrospective history; sepsis criteria and cerebral-injury timing unconfirmed.
26 Nov 2024	Bowel perforation reportedly identified on abdominal CT.	Exploratory laparotomy and ileostomy.	Retrospective history; primary imaging/operative reports unavailable.
5–7 Dec 2024	Serum/CSF sampled on 5 Dec; neuroimmunological results issued on 6–7 Dec.	Targeted antibodies negative; detailed results in Supplementary Table 2.	Original laboratory reports; routine CSF/infectious studies unavailable.
2 Jan 2025	Drowsy but arousable; severe hypertonia, wrist contractures and foot drop persisted.	Admission for neurological evaluation and supportive/rehabilitation care.	Admission examination; cerebral etiology remained uncertain.
4–8 Jan 2025	Transient simultaneous tachycardia, raised blood pressure, tachypnea, fever, sweating and abnormal posturing described.	PSH considered on 4 Jan; consultant judged the symptoms consistent with PSH on 8 Jan; multimodal symptom control.	Dated notes; clinical scoring date unconfirmed; no EEG capture of a typical event available.
13–20 Jan 2025	Pulmonary infection and *E. coli* urine culture reported; fewer paroxysms noted on 15 Jan.	Piperacillin-tazobactam and concurrent multimodal care, including intravenous valproate.	Dated notes; qualitative improvement during combined care, with no isolated drug effect established.
21–24 Jan 2025	More episodes noted on 23–24 Jan after combined medication reduction.	Analgesic/sedative and antiseizure treatment reduced on 21–22 Jan and restored after recurrence. Empirical piperacillin–tazobactam resumed on 23 Jan.	Dated notes; PSH recurrence, seizures and withdrawal remained unresolved.
26–31 Jan 2025	Reduced paroxysm intensity/duration described; no obvious rigidity episode noted on 31 Jan.	Multimodal symptom treatment, infection-directed care and rehabilitation continued. The 31 Jan note reported antibiotics stopped the previous day.	Dated notes; no numerical episode frequency or severity measurements.
1–5 Feb 2025	Febrile illness with hypotension and leukocytosis on 1–2 Feb; *Candida parapsilosis* urine culture reported on 5 Feb.	Renewed infection-directed care and subsequent antifungal treatment.	Dated notes; final blood culture results unavailable; findings not used as PSH scoring peaks.
7 and 12 Feb 2025	Clearer communication and improved tone; no obvious rigidity episodes at these assessments; contractures and foot drop persisted.	Intrahospital transfer to our rehabilitation department on 12 Feb.	Progress/transfer notes from 7/12 Feb. MRI images are dated 20 Feb. Formal functional scores unavailable.

On January 2, the patient was drowsy but arousable and answered simple questions in a low-volume voice. Temperature was 36.9 °C, blood pressure 112/77 mmHg, heart rate 117 beats/min, and respiratory rate 16 breaths/min. He followed simple head/facial commands but could not move his limbs voluntarily. Examination showed marked hypertonia, bilateral wrist contractures, foot drop, mild neck resistance, equal reactive pupils, and absent bilateral Babinski signs.

The supplied brain magnetic resonance imaging (MRI) images are dated February 20, 2025. Available sequences included sagittal T2-weighted and axial T1-weighted, T2-weighted, fluid-attenuated inversion recovery (FLAIR), diffusion-weighted imaging (DWI), and apparent diffusion coefficient (ADC) series. Bilateral symmetric periventricular and deep white matter FLAIR/T2 hyperintensities showed corresponding DWI hyperintensity without definite ADC reduction (Figure 1). Earlier brain imaging for comparison and information on contrast enhancement were unavailable, so the onset and evolution of these abnormalities could not be determined. Toxic-metabolic, hypoxic-ischemic, inflammatory, and other critical illness-associated processes remain possible explanations.

**FIGURE 1:**
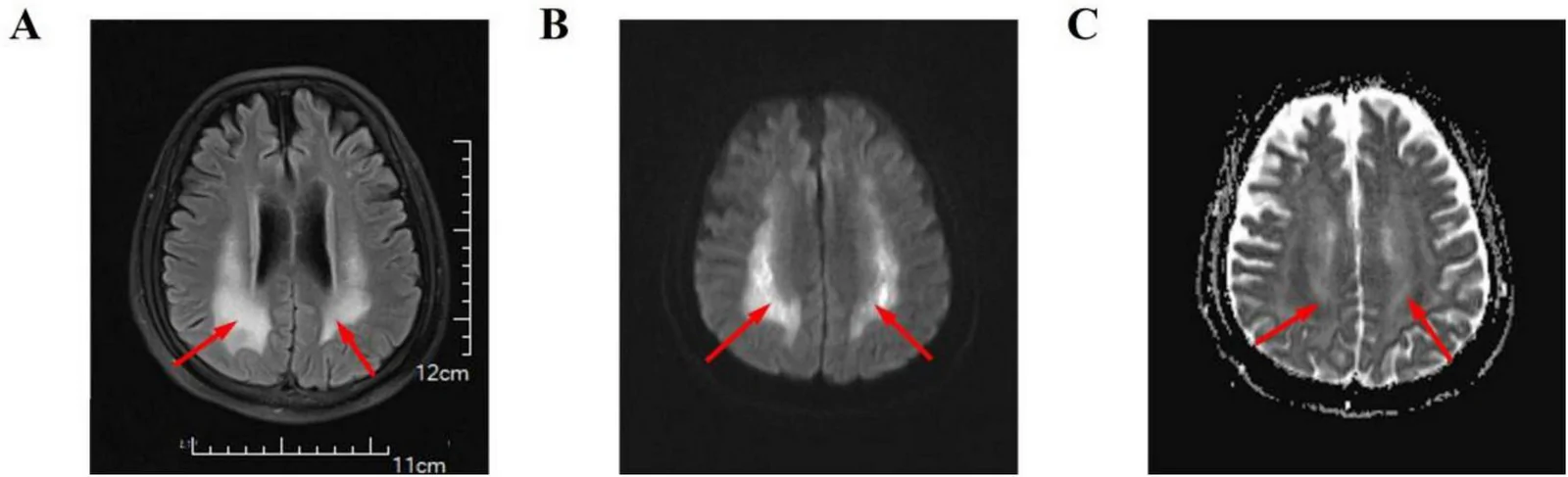
Magnetic resonance imaging (MRI) findings during diagnostic evaluation. **(A)** Bilateral periventricular/deep white matter FLAIR hyperintensities. **(B)** Corresponding DWI hyperintensity. **(C)** No definite ADC reduction. The images are dated February 20, 2025. These nonspecific findings do not establish the onset or cause of the white matter abnormalities or a specific PSH etiology.

Original neuroimmunological reports documented serum and cerebrospinal fluid (CSF) sampling on December 5, 2024, with results issued on December 6–7. Tested serum/CSF antibodies against AQP4, MOG, GFAP, NMDAR, AMPAR, GABABR, LGI1, and CASPR2 were negative; antibody testing used indirect immunofluorescence with tissue- and cell-based assays. Oligoclonal bands had a type 4 pattern, with an IgG index of 0.67 and synthesis rate of 0.48 mg/24 h (Supplementary Table 2). These findings do not establish a defined antibody-mediated disorder or exclude seronegative/infectious disease. Routine CSF cell count, total protein, glucose, opening pressure, and infectious culture/PCR results remained unavailable.

On January 4, clinicians documented frequent, transient, simultaneous tachycardia, increased blood pressure, tachypnea, fever, profuse sweating, and abnormal posturing, and considered PSH. Notes over January 5–8 described recurring rigidity with tachycardia, tachypnea, and sweating that eased after symptomatic treatment. The January 8 ICU consultation recorded heart-rate fluctuations of 80–160 beats/min during ongoing analgesic/sedative infusions and described episodic worsening of baseline rigidity with predominantly cranial sweating. Exact daily counts, duration in minutes, stimulus relationships, post-event state, active assessment of parasympathetic features, and synchronized episode-specific vital-sign peaks were not recorded.

Butorphanol and esmolol infusions were documented on January 4; eperisone was added on January 5. Additional medication-order summaries listed oral bromocriptine 2.5 mg three times daily, baclofen 10 mg three times daily, gabapentin 600 mg three times daily, and sodium valproate 500 mg once daily. These are reported prescriptions; actual administration and complete exposure durations could not be independently verified. The summaries also contained intravenous dexmedetomidine and propofol orders, but execution could not be confirmed. Verified intravenous doses, delivery rates, and exact valproate infusion initiation/transition dates were unavailable (Supplementary Table S3).

January 3 investigations showed hemoglobin 85 g/L, platelets 510 × 10^9^/L, procalcitonin 0.382 ng/mL, D-dimer 1.06 mg/L, creatinine 62 μmol/L, bilirubin 5.7 μmol/L, and creatine kinase (CK) 32 U/L. Interleukin-6 (IL-6) was 20.6 pg/mL and procalcitonin 0.177 ng/mL on January 15. Selected dated trends are presented in Supplementary Table S1. The January 3 PaO_2_ of 125 mmHg on FiO_2_ 0.40 and lactate of 0.8 mmol/L describe that measurement only. A later inpatient summary mentioned hypoxemia, and February 2 documented hypotension; the earlier ICU hypoxic, hemodynamic, and metabolic course remains unknown.

Later infections were documented separately from the November postoperative event. On January 13, chest radiography was reported as showing pulmonary infection, with a white blood cell count (WBC) of 12.57 × 10^9^/L and C-reactive protein (CRP) of 36.4 mg/L; piperacillin–tazobactam was started. A January 14 note reported an E. coli urine culture. CRP fell to 12.1 mg/L on January 19 and antibiotics had been stopped by January 20, although paroxysms persisted. The January 23 note recorded resumption of empirical piperacillin–tazobactam for recurrent fever. The January 31 note reported discontinuation the previous day. On February 1–2, fever to 40 °C, heart rate 130–160 beats/min, respiratory rate approximately 30 breaths/min, blood pressure as low as 85/47 mmHg, and WBC 30.92 × 10^9^/L prompted renewed infection-directed treatment. Blood and catheter-blood cultures were collected, but final results were unavailable. A February 5 note reported *Candida parapsilosis* in urine and antifungal treatment. Candiduria alone does not establish invasive infection. These later findings do not retrospectively confirm November sepsis and were not used as PSH scoring peaks.

On January 8, an ICU consultant judged the symptoms consistent with PSH, which remained among the February 12 transfer diagnoses. A clinician-completed PSH-AM assessment during care recorded CFS 15, DLT 9 and total 24, classified as probable PSH (Baguley et al., 2014). The assessment date and raw episode measurements were unavailable. Retaining the clinician-recorded CFS grades and reviewing DLT against the available notes yielded five supported criteria. Six unresolved or unsupported items received 0 for insufficient evidence, not confirmed absence. Recorded CFS 15 plus reviewed DLT 5 yields 20, retaining probable PSH (Table 2). This calculation uses the recorded severity grades rather than independently reconstructed measurements. Later infection-related maxima were not substituted.

**TABLE 2:** Clinician-recorded CFS grades and conservative PSH-AM reassessment.

PSH-AM component	Scoring source / recoverable evidence	Score
Clinical Feature Scale (CFS)		
Heart rate	Reported grade 3 (threshold ≥ 140/min); Jan 8 range 80–160/min during infusions; event attribution unconfirmed.	3
Respiratory rate	Reported grade 3 (threshold ≥ 30/min); corresponding episode values/date unavailable.	3
Systolic blood pressure	Reported grade 1 (140–159 mmHg); corresponding episode values/date unavailable.	1
Body temperature	Reported grade 2 (38.0–38.9 °C); corresponding episode values/date unavailable; later infection peaks not used to reconstruct CFS.	2
Sweating	Reported grade 3; profuse/cranial sweating described; original intensity assignment not independently revalidated.	3
Posturing	Reported grade 3; baseline rigidity and episodic worsening documented; original intensity assignment not independently revalidated.	3
Recorded CFS subtotal	CFS grades retained from the clinician’s assessment during care (original CFS 15, DLT 9, total 24).	15
Diagnostic Likelihood Tool (DLT)		
Features occur simultaneously	Explicitly documented in Jan 4 clinical assessment.	1
Episodes are paroxysmal	Transient recurrent events documented Jan 4–8.	1
Over-reactivity to normally non-painful stimuli	No case-specific trigger relationship documented.	0
Features persist ≥ 3 consecutive days	Recurring events in consecutive Jan 5–8 notes.	1
Features persist ≥ 2 weeks after brain injury	Nov 14 is reported dysfunction onset; a brain-injury date is not established.	0
Persist despite treatment of alternatives	Paroxysms persisted during infection-directed treatment and IV valproate; Jan 20 notes describe improvement of infection. This does not exclude seizures or infection.	1
Medication given to reduce sympathetic features	Butorphanol/esmolol recorded Jan 4; additional agents by Jan 9–10.	1
≥ 2 episodes daily	Frequent/intermittent descriptions without reliable daily counts.	0
Absence of parasympathetic features	No evidence of active systematic assessment.	0
Absence of another presumed cause	Seizures and other contributors remain unresolved.	0
Antecedent acquired brain injury	Encephalopathy and MRI abnormalities suggest dysfunction but do not establish a timed acquired injury.	0
Reviewed DLT subtotal	Five supported items. Six items score 0 for insufficient evidence, not confirmed absence.	5
Reassessed total PSH-AM score	Recorded CFS 15 + reviewed DLT 5. Severity grades were not independently reconstructed.	20
PSH-AM category	Score ≥ 17 using the recorded CFS. Seizures and cerebral etiology remain unresolved.	Probable PSH

The cause of extensive arterial thrombosis remained unresolved. A January 3, 2025, transthoracic echocardiogram reported a left ventricular ejection fraction of 64% with mild mitral/tricuspid regurgitation. Vascular ultrasonography on January 3 and February 13 showed persistent left superficial femoral and popliteal arterial occlusion. These findings do not establish the original thrombosis mechanism or exclude a cardiac embolic source. Specific antiphospholipid, thrombophilia, vasculitic, and hematological etiological results remained unavailable. No mechanistic link from systemic thrombosis to the paroxysmal syndrome can be inferred.

The January 15 progress note documents intravenous valproate use and fewer episodes during combined therapy. The exact initiation date could not be established from the available records. During January 21–24, reduction of analgesic/sedative and antiseizure medications was followed by more episodes, prompting restoration of prior doses. Notes from January 26–February 1 described reduced frequency, intensity, and duration without numerical measurements. Butorphanol, remimazolam, valproate, and esmolol infusions were listed on February 3. Intravenous treatment was reduced in early February while oral treatment continued; the precise valproate infusion stop/transition date could not be confirmed. Improvement occurred during combined pharmacological, infection-directed, supportive, and rehabilitation care; neither a single-drug effect nor an ictal mechanism can be established. Systematic drug-specific tolerability and adverse-event assessments were unavailable.

By February 7, the patient was alert, answered clearly, followed simple head/facial commands, and had no obvious rigidity episodes, but remained unable to move his limbs. On February 12, he was transferred to the rehabilitation department of our hospital. The transfer record retained PSH among the clinical diagnoses and described improved tone, no fever or obvious rigidity episodes, and persistent bilateral wrist contractures and foot drop. No documented GCS, mRS, GOS-E, or CRS-R was available, and no retrospective score was inferred. Subsequent medication-order summaries included entries through March, but provided no quantitative episode or functional outcome assessments. The authors were subsequently informed of discharge to a specialized rehabilitation hospital; this separately reported destination, its date, and receiving-hospital clinical/imaging follow-up could not be verified from the reviewed records. A direct account of the patient’s treatment experience was not recorded.

## 3 Discussion

The distinctive clinical setting comprised extensive arterial thrombosis, vascular intervention, suspected postoperative sepsis, prolonged encephalopathy, and bilateral white matter abnormalities. Serial observations of simultaneous paroxysmal sympathetic and motor features informed the treating-team PSH diagnosis. The conservative PSH-AM total remained in the probable category, supporting the syndromic interpretation while cerebral etiology remained uncertain.

The longitudinal course adds context to published adult non-traumatic PSH reports, which include patients with hypoxic-ischemic encephalopathy and meningoencephalitis (Chahar et al., 2026). In this case, paroxysms persisted as January infection markers improved and antibiotics were temporarily stopped by January 20. Physiologic overlap between infection and PSH has also been studied after traumatic brain injury (Salasky et al., 2024). Infection coexisted here and could account for some later fever and hemodynamic abnormalities, supporting continued assessment of both processes.

Models of PSH propose impaired descending inhibition of sympathetic and motor circuits (Louraoui et al., 2022; Moffet et al., 2026). These models provide context for considering cerebral dysfunction in this patient, but the imaging does not establish a specific autonomic-network lesion. Neither systemic thrombosis nor suspected postoperative sepsis can be assigned a causal role in PSH from the available evidence.

The December 2024 neuroimmunological results and the MRI images dated February 20, 2025, inform the cerebral differential at different time points. Negative targeted antibodies reduced support for tested antibody-defined disorders but did not exclude seronegative disease. The type 4 oligoclonal-band pattern did not establish CNS-restricted inflammation. Toxic-metabolic, hypoxic-ischemic, inflammatory, and other critical illness-associated processes remain possible cerebral etiologies. These investigations inform etiological assessment rather than establish the PSH diagnosis.

The differential assessment in Table 3 separates competing or coexisting causes of paroxysms from possible etiologies of cerebral dysfunction. Repeated simultaneous autonomic and motor changes favored the clinical PSH interpretation. Earlier seizure-like events, absent EEG event capture, and improvement during valproate-containing combined therapy leave an ictal contribution unresolved. Incomplete exposure histories also limit assessment of medication syndromes and withdrawal.

**TABLE 3:** Competing diagnoses and possible causes of cerebral dysfunction.

Diagnosis / role	Supporting findings	Counterpoints / available assessment	Residual uncertainty
Epileptic seizures (mimic/coexistence)	Earlier upward eye deviation and seizure-like events; fewer events during IV valproate and combined care.	Repeated simultaneous autonomic–motor episodes supported the treating-team PSH diagnosis; not specific.	No EEG report or event capture; concurrent seizures unresolved.
Ongoing infection / suspected sepsis (coexistence/mimic)	Jan pulmonary infection / E. coli urine; Feb fever, hypotension, leukocytosis; candiduria.	Paroxysms persisted during improvement of Jan infection markers.	Later fever and hemodynamic abnormalities coincided with documented infection. November sepsis criteria unrecoverable.
CNS infection (cerebral etiology/mimic)	Fever, encephalopathy and mild neck resistance.	Targeted neuroimmune antibodies do not assess CNS infection.	Routine CSF, culture and pathogen PCR unavailable; not excluded.
Metabolic / toxic / hypoxic processes (cerebral etiology)	Prolonged critical illness; alcohol history; nonspecific white matter findings.	Selected Jan gases/electrolytes do not identify a single cause.	Earlier oxygenation, perfusion and exposure history incomplete; normal later samples do not exclude prior insults.
Neuroleptic malignant syndrome (mimic)	Fever, rigidity and altered consciousness overlap.	No temporal dopamine-antagonist exposure established; CK 32 U/L on Jan 3 is a later single sample.	Outside-hospital dopamine-antagonist exposure remains undocumented.
Serotonin syndrome (mimic)	Autonomic and motor features overlap.	No linked serotonergic exposure established.	Exposure chronology and systematic clonus/hyperreflexia assessment unavailable.
Alcohol / medication withdrawal (mimic/coexistence)	Longstanding alcohol use. More episodes during combined medication reduction in January.	January recurrence followed medication reduction and may reflect PSH, seizures or medication withdrawal.	Alcohol cessation timing was not recorded. Sedative/analgesic exposure and taper details were incomplete.
Autoimmune / inflammatory disease (cerebral etiology)	Encephalopathy and outside-hospital concern for CNS demyelination.	Tested serum/CSF antibodies negative; type 4 OCB and IgG indices do not define a specific disorder.	Seronegative or other inflammatory disease remains possible. Earlier imaging and routine CSF results unavailable.

PSH management commonly uses combined symptom control and supportive measures (Kamate et al., 2025; Nalliah et al., 2026). The treatment course showed fewer episodes during combined care, recurrence during medication reduction, and subsequent improvement after treatment adjustment. These observations describe a clinical course without identifying an effective individual drug or distinguishing PSH recurrence from seizures or withdrawal. By the rehabilitation transfer, clearer communication and reduced tone coexisted with persistent severe limb motor impairment, contractures, and foot drop. This course highlights the need to assess symptom control and functional recovery separately.

The principal limitations are retrospective outside-hospital history, unavailable earlier brain imaging and uncertain cerebral etiology, incomplete postoperative sepsis documentation, and absent EEG event capture. Assessment timing, raw episode measurements, and daily counts were unavailable; the revised PSH-AM retains clinician-recorded CFS grades rather than independently reconstructed severity measurements. Medication summaries provide prescription doses, but complete administration records, infusion rates, exposure durations, and systematic adverse-event assessments were unavailable. Thrombosis etiology remained unresolved. Routine CSF/infectious studies, formal functional scores, and long-term neurological/radiological outcomes were unavailable.

## 4 Conclusion

This case illustrates probable PSH recognized during vascular and postoperative critical illness with encephalopathy and bilateral white matter abnormalities. Repeated simultaneous autonomic–motor episodes and a conservative PSH-AM total in the probable range supported the treating-team diagnosis. Improvement during multimodal care was accompanied by persistent severe motor impairment and rehabilitation needs. Serial episode assessment should accompany evaluation of infection and seizures, while symptom control and functional recovery are assessed separately. Cerebral etiology remained uncertain, and coexisting seizures could not be excluded.

## Data Availability

The original contributions presented in this study are included in this article/Supplementary material, further inquiries can be directed to the corresponding author.
