# Supplementary material for: Case Report: Probable paroxysmal sympathetic hyperactivity during vascular and postoperative critical illness with bilateral white matter abnormalities
Source: Front Neurosci. 2026 Oct 29;20:1935805. doi: 10.3389/fnins.2026.1935805 (PMC13755225; doi:10.3389/fnins.2026.1935805)
Supplement: Supplementary file 1 [file Table_1.docx]

**Supporting Information**

**Case Report: Probable Paroxysmal Sympathetic Hyperactivity During Vascular and Postoperative Critical Illness With Bilateral White Matter Abnormalities**

Mozhen Li^1^, Yujie Wei^1^, Zhenhua Cai^1^, Lusheng Wang^2^, Shengwen Guo^1^, Jifei Cao^1^, Heng Tang^2^, Lei Wang^1*^

^1^Intensive Care Unit, Wanbei Coal Electric Group General Hospital, Suzhou, Anhui 234000, China, ^2^Central Laboratory, Wanbei Coal Electric Group General Hospital, Suzhou, Anhui 234000, China

*Corresponding author: Lei Wang, E‑mail: wl3982795@163.com

**Supplementary Table S1. Selected dated laboratory and infection findings**

| **Date** | **Findings** | **Interpretive limits** |
| --- | --- | --- |
| 3 Jan 2025 | Hb 85 g/L; platelets 510 × 10⁹/L; WBC 8.47 × 10⁹/L; PCT 0.382 ng/mL; D-dimer 1.06 mg/L; creatinine 62 µmol/L; bilirubin 5.7 µmol/L; CK 32 U/L. PaO_2_ 125 mmHg on FiO_2_ 0.40; lactate 0.8 mmol/L. | Single sample after admission to our hospital; cannot exclude earlier cerebral insults or drug syndromes. |
| 6–7 Jan | PCT 0.557 ng/mL (6 Jan); CRP 30.9 mg/L (7 Jan). | Inflammatory markers alone do not establish sepsis. |
| 13–14 Jan | WBC 12.57 × 10⁹/L and CRP 36.4 mg/L (13 Jan); pulmonary infection reported on radiograph. E. coli urine culture reported in 14 Jan note. | Culture sampling date, susceptibility report and relation to organ dysfunction unavailable. |
| 15 Jan | IL-6 20.6 pg/mL; PCT 0.177 ng/mL; D-dimer 0.89 mg/L. | Later inflammation does not confirm the November postoperative diagnosis. |
| 19 Jan | CRP 12.1 mg/L. | Episodes persisted despite improvement in infection indicators. |
| 27 Jan | TAT 3.94 ng/mL; PIC 0.79 µg/mL; thrombomodulin 10.05 TU/mL; t-PAIC 1.34 ng/mL; D-dimer 0.59 mg/L. | These markers are not a complete etiological thrombophilia work-up. |
| 1–2 Feb | Fever to 40 °C; HR 130–160/min; RR approximately 30/min; BP 97/48 then 85/47 mmHg. WBC 30.92 × 10⁹/L and lactate 0.9 mmol/L (2 Feb). | Treated as recurrent infection; these findings were not used to reconstruct the clinician-recorded CFS grades. |
| 3–5 Feb | WBC 28.40 × 10⁹/L (3 Feb); CRP 117 mg/L (4 Feb); urine Candida parapsilosis reported 5 Feb. | Blood/catheter-blood culture final reports unavailable. Candiduria does not establish invasive candidiasis. |

**Supplementary Table S2. Reported neuroimmunological investigations**

| **Test** | **Serum** | **CSF** |
| --- | --- | --- |
| Anti-AQP4 IgG | Negative | Negative |
| Anti-MOG IgG | Negative | Negative |
| Anti-GFAPα IgG | Negative | Negative |
| Anti-GFAPε IgG | Negative | Negative |
| Anti-NMDAR IgG | Negative | Negative |
| Anti-AMPAR1 IgG | Negative | Negative |
| Anti-AMPAR2 IgG | Negative | Negative |
| Anti-GABABR IgG | Negative | Negative |
| Anti-LGI1 IgG | Negative | Negative |
| Anti-CASPR2 IgG | Negative | Negative |
| Oligoclonal bands | Corresponding serum/CSF bands reported | Type 4 |
| IgG index | — | 0.67 |
| IgG synthesis rate | — | 0.48 mg/24 h |
| Routine CSF cell count, total protein, glucose; opening pressure | Not applicable | Unavailable |
| CSF infectious culture / pathogen PCR | Not applicable | Unavailable |

**Supplementary Table S3. Documented treatment changes and qualitative response**

| **Date / documentation** | **Treatment / route** | **Response and limitations** |
| --- | --- | --- |
| 4 Jan | Butorphanol and esmolol infusions documented in the progress note. | Progress-note documentation of treatment; verified IV doses and infusion rates unavailable. |
| 5 Jan | Eperisone added (tablets subsequently listed). | Recurring rigidity/autonomic episodes continued; symptomatic relief described. |
| January oral-order summaries | Bromocriptine 2.5 mg three times daily; baclofen 10 mg three times daily; gabapentin 600 mg three times daily; sodium valproate 500 mg once daily, all oral. | Prescription entries do not establish a simultaneous regimen; administration and complete start/stop chronology unverified. Overlapping entries are not added together. |
| 15 Jan | IV valproate and other therapies ongoing; exact initiation date not securely established. | Fewer episodes described without daily counts; an isolated valproate effect or ictal mechanism cannot be determined. |
| 21–24 Jan | Analgesic/sedative and antiseizure treatment reduced; prior doses restored after recurrence. | More episodes during combined reduction; not attributable to a single agent or definitive withdrawal. |
| 26 Jan–1 Feb | Combined symptom-directed treatment continued; gradual reduction of IV treatment planned/recorded. | Frequency, intensity and duration improved qualitatively; numeric series unavailable. |
| 3 Feb | Continuous IV butorphanol, remimazolam, valproate and esmolol explicitly documented, with dose reduction described. | Concurrent febrile infection episode; drug-specific adverse-event attribution unavailable. |
| Early February | Reduction of IV treatment while oral symptom-directed treatment continued; exact valproate infusion stop/transition date unconfirmed. | No obvious rigidity episodes in later bedside notes; no quantitative frequency or severity series. |
| Throughout inpatient course | Nutrition, electrolyte management, anticoagulation and pulmonary/skin care; rehabilitation and passive joint exercises. | These measures and infection treatment overlapped pharmacological symptom control. |
| Infection-directed care | Piperacillin–tazobactam started 13 Jan and stopped by 20 Jan. Resumption recorded 23 Jan; the 31 Jan note reported discontinuation the previous day. Renewed infection-directed care in early February. Amphotericin B cholesteryl sulfate complex and bladder irrigation recorded 5 Feb. | Antimicrobial doses, complete administration dates and systematic toxicity assessment unavailable. |
| After 12 Feb rehabilitation transfer | Later oral medication-order summaries include entries through March. | Prescriptions do not establish delivered exposure, ongoing PSH, or neurological recovery; functional and episode follow-up unavailable. |
